# Bioprocess development for bacterial cellulose biosynthesis by novel *Lactiplantibacillus plantarum* isolate along with characterization and antimicrobial assessment of fabricated membrane

**DOI:** 10.1038/s41598-022-06117-7

**Published:** 2022-02-09

**Authors:** Ahmed K. Saleh, Hamada El-Gendi, Nadia A. Soliman, Waleed K. El-Zawawy, Yasser R. Abdel-Fattah

**Affiliations:** 1 Cellulose and Paper Department, National Research Centre, El-Tahrir St., Dokki, Giza, Egypt; 2 Bioprocess Development Department, Genetic Engineering and Biotechnology Research Institute, City of Scientific Research and Technological Applications, Alexandria, Egypt

**Keywords:** Biological techniques, Biotechnology, Evolution, Microbiology

## Abstract

Bacterial cellulose (BC) is an ecofriendly biopolymer with diverse commercial applications. Its use is limited by the capacity of bacterial production strains and cost of the medium. Mining for novel organisms with well-optimized growth conditions will be important for the adoption of BC. In this study, a novel BC-producing strain was isolated from rotten fruit samples and identified as *Lactiplantibacillus plantarum* from *16S* rRNA sequencing. Culture conditions were optimized for supporting maximal BC production using one variable at a time, Plackett–Burman design, and Box Behnken design approaches. Results indicated that a modified Yamanaka medium supported the highest BC yield (2.7 g/l), and that yeast extract, MgSO_4_, and pH were the most significant variables influencing BC production. After optimizing the levels of these variables through Box Behnken design, BC yield was increased to 4.51 g/l. The drug delivery capacity of the produced BC membrane was evaluated through fabrication with sodium alginate and gentamycin antibiotic at four different concentrations. All membranes (normal and fabricated) were characterized by scanning electron microscope, Fourier transform-infrared spectroscopy, X-ray diffraction, and mechanical properties. The antimicrobial activity of prepared composites was evaluated by using six human pathogens and revealed potent antibacterial activity against *Escherichia coli, Klebsiella pneumoniae, Staphylococcus aureus*, and *Streptococcus mutans*, with no detected activity against *Pseudomonas aeruginosa* and *Candida albicans*.

## Introduction

Cellulose is the most widely distributed biodegradable and renewable biopolymer as the predominant component of plant biomass. Cellulose can be extracted from various sources including plants (plant cellulose [PC]); it iswidely applied in textile and food industries^1^. Bacterial cellulose (BC) is another type of cellulose produced from bacteria^2^. BC is an extra-polysaccharide composed of glucose units connected via β-(1-4)-glycosidic bonds forming glucan chains [(C_6_H_10_O_5_)n]^3,4^, first reported by Brown^7^ from *Acetobacter xylinus* as a gelatinous mat at the air–liquid interface during acetic acid fermentation^5–7^. BC polymers have diverse commercial applications, including food packaging, food products, biomaterials, biomedical products, water treatment processes, electronics, and cosmetics^6,8–13^. BC fabricated with other gelling materials, such as sodium alginate (SA)^14^, chitosan^15^, polyethylene glycol^16^ and gelatin^17^, can also be used as a carrier for bioactive compounds, such as antibiotics. Consequently, wound dressings are emerging as a key application for BC. BC shows advantages over other types of cellulose with respect to its ultrafine network purity (devoid of hemicelluloses, lignin, pectin, and wax), high capacity for water retention, porosity, crystallinity, degree of polymerization, biocompatibility, biodegradability, and useful mechanical properties (Young’s modulus value, tensile strength, compressibility, and elongation)^18–21^. Thus far, the high cost of BC production represents a great challenge to the wide commercial adoption of BC.

**Figure 1:**
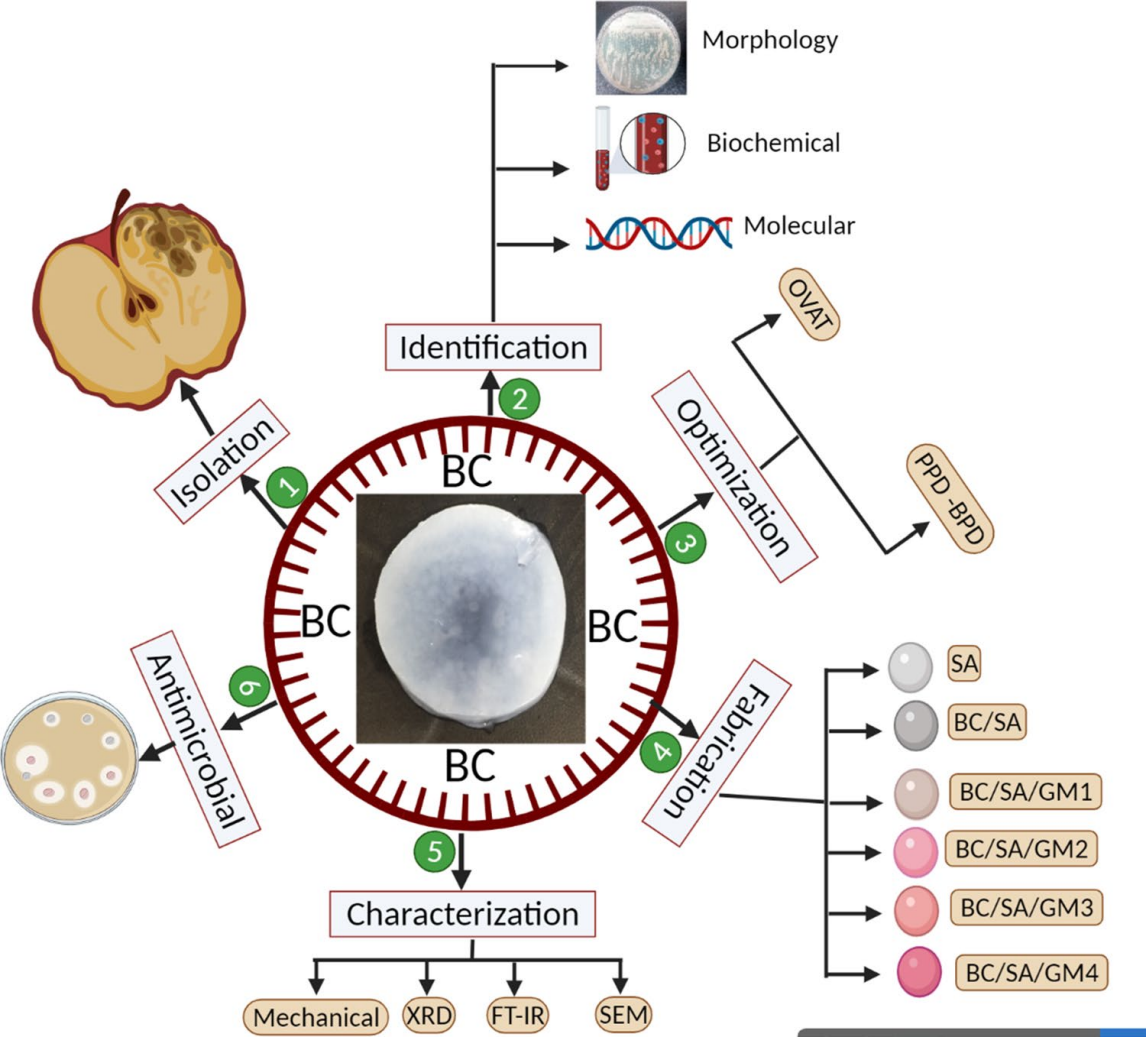
Schematic characterize the procedures of the present work.

Gram-negative *Komagataeibacter* sp. that belong to a group of acetic acid bacteria are considered to be the model organism for BC production and have been extensively described in the literature^6,22–25^. By contrast, BC production from Gram-positive bacteria has been reported from only a few species such as *Lactobacillus* sp.^26,27^, *Bacillus* sp.^28,29^, *Leifsonia* sp.^30,31^, and *Rhodococcus* sp.^32^. Identifying other high yielding strains would enhance the commercial application of BC in different sectors. Optimizing the production, including the composition of the medium and parameters for cultivation conditions is critical for cost reduction. Various optimization techniques can be applied to enhance BC yields, including one variable at a time (OVAT) methods or statistical experimental designs (SED)^22,28,33^. The OVAT technique is a conventional optimization approach wherein variables are held constant and only one variable is altered. Optimization of BC production through an OVAT approach has been extensively used to study medium compositions, pH, incubation temperature, inoculum size, and incubation period^33–36^. Although simple, the OVAT approach has many drawbacks as it can be highly time-consuming and labor intensive, especially when screening multiple variables. Moreover, it ignores interactions between variables^37^. The application of statistical experimental designs can enhance the efficiency of process optimization^38^. SED involves a multistep approach, wherein a vast number of variables are simultaneously studied in the same experiment using screening designs such as Plackett–Burman design (PBD). After this screening stage, the precise role of each variable on process yield and the nature of variable interactions can be determined through optimization designs, such as Box Behnken design (BBD), that determine the optimum level for the most important variables^39–41^. Recently, SED have been successfully applied to optimize the medium composition and cultivation conditions for BC production from a number of bacterial strains^22,28,42,43^. The current study focused on the state-of-the-art of BC production by a true probiotic bacterial isolate for medical application. Different bioprocess optimization strategies were followed for maximization of BC yields. Furthermore, the obtained BC through applying the optimized medium was fabricated with SA at different concentrations of gentamycin (GM) for formation a new drug delivery system as wound dressing materials for inhibition the microbial growth (Fig. 1). Hence, the work novelty can be summarized in two points: Firstly, using of probiotic isolate for BC production namely *L﻿*. *Plantarum* this is considered the first reported BC production by this species and at the same moment following up different strategies for maximizing the production using a low cost medium. Secondly, a trial to construct new drug delivery system through BC fabrication which is highly suitable, where BC derived from a safe probiotic strain is acceptable for medical application. This characteristic is very important for medical applications and opens the door for further application in the near future. Our study aimed to identify novel BC-producing bacteria and establish conditions for culture. We identify the Gram-positive *Lactiplantibacillus plantarum (L. plantarum* AS.6) strain as an effective BC-producing strain. Medium compositions and culture conditions affecting the BC production were evaluated and properties of the resulting BC membrane were characterized. The antimicrobial activity of the resulting BC membrane fabricated to SA and GM was also evaluated.

## Results and discussions

### Isolation, screening, and identification of BC-producing strain

**Figure 2:**
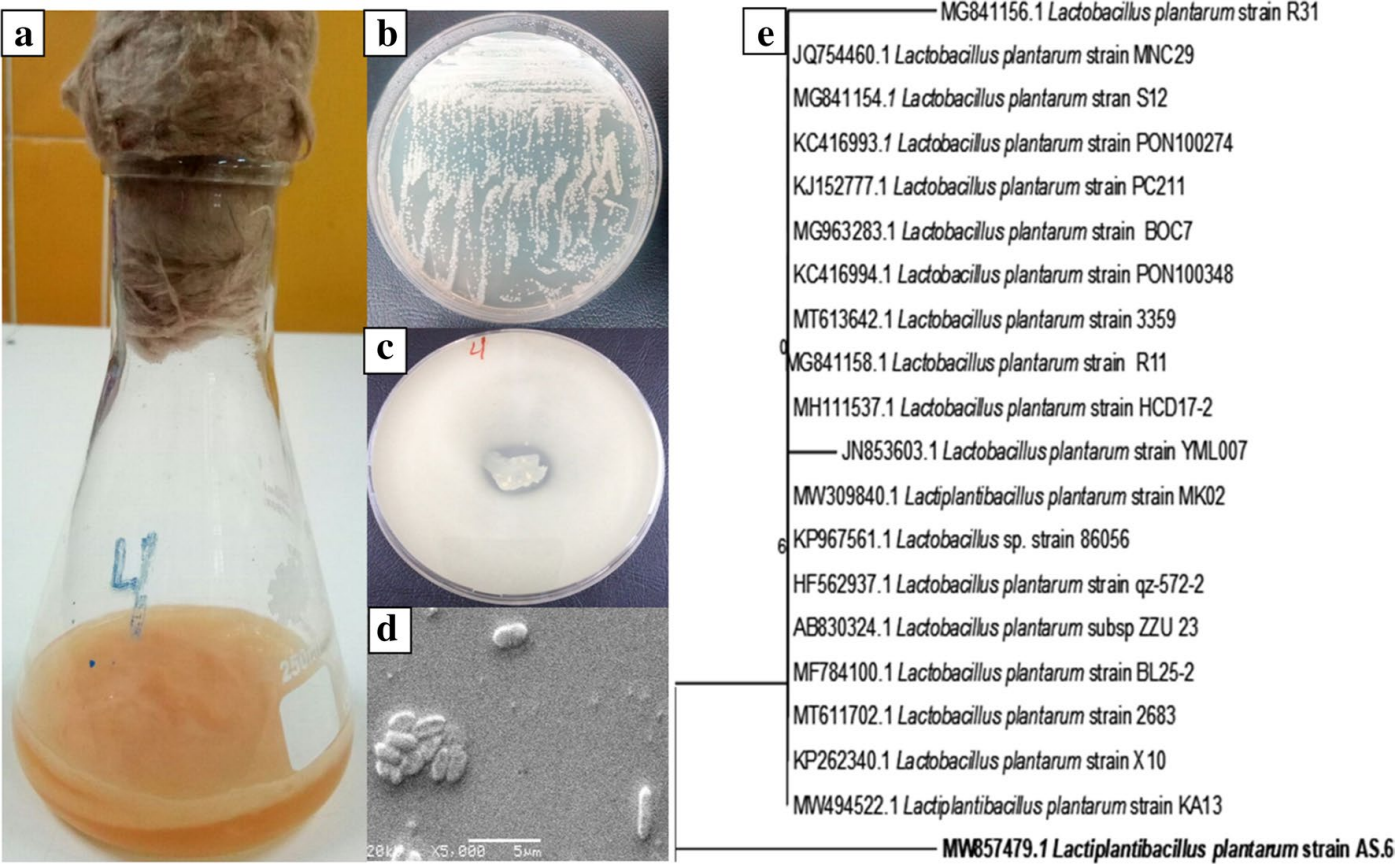
Eye observation of BC (**a**), purified and separate colony (**b**), acid production (**c**) and SEM (**d**) and phylogenetic tree based on 16S rDNA sequences (**e**) of *L. plantarum* AS.6.

Rotten fruits, vegetables, and vinegar are frequently used for the isolation of BC-producing bacteria. Flasks contained MHS media were inoculated with rotting samples after the addition of yeast/mold inhibitors (Nystatin). After 7 days of incubation, the flask with rotten apple was reported as a positive result for BC production as the formation of a creamy white gelatinous mat-like structure (BC pellicle) at the air–liquid interface was observed that was not destroyed by vortexing or high-speed centrifugation. The isolates were purified by serial dilution to obtain 58 different samples and screened for BC production using HS media. Only one isolate (number 4) was selected as a BC producer under static conditions (Fig. 2a). This isolate was stored at 4 °C in an HS agar slant for further studies. Similarly, a few studies have reported that BC producer strains may be isolated from rotten apples such as *Gluconacetobacter hansenii* PJK^44^, *Enterobacter amnigenus* GH-1^45^, and *Gluconacetobacter* sp.^46^. In these studies, identification of the BC-producing isolate was executed mainly through molecular identification or morphological and biochemical characterization. To characterize the identity of the bacterial strain in isolate 4, analysis of the *16S rDNA* (partial sequence) revealed that the selected strain (No. 4) showed 96.15% similarity to the *Lactobacillus plantarum* strain X10 (KP262340.1) sequence. A Phylogenetic tree was designed using the Clustal X program (Fig. 2e), showing that the isolate AS.6 is more related to *Lactiplantibacillus plantarum* KA13 (MW494522.1) by 96.28%. The AS.6 strain *16S* rRNA gene sequence was deposited in the GenBank under the accession number (MW857479.1), under the name of *Lactiplantibacillus plantarum* strain AS.6 (*L. plantarum* AS.6). The investigated bacterium *L. plantarum* AS.6 isolated from rotten apple was subjected to morphological characterization through purified colony analysis (Fig. 2b), scanning electron microscope (SEM) (Fig. 2d), and colony characterization (Table 1). The biochemical characteristics for AS.6 strain were investigated using a commercially available Oxoid kit (Microbact GNB 24E kit) with reference to Bergey’s Manual of Systematic Bacteriology as shown in Table 1. *L. plantarum* AS.6 was screened as being an acid-producing bacteria by the visualization of a clear zone around the bacterial colony due to dissolution of CaCO_3_ in GEY agar plates supplemented with 0.3% CaCO_3_ as showed in Fig. 2c.

**Table 1:** Morphological and biochemical characteristics of *L. plantarum* AS.6.

Morphological		Biochemical							
Colony	Characterization	Test	R	Test	R	Test	R	Test	R
Configuration	Bacilli	Motility	+	Xylose	+	Gelatin	−	Arabinose	+
Margin	Entire	Nitrate	−	ONPG	−	Malonate	−	Adonitol	−
Surface	Smooth	Lysine	−	Indole	−	Inositol	−	Raffinose	−
Pigment	Cream	Ornithine	−	Urease	−	Sorbitol	−	Salicin	−
Gram reaction	Positive	H_2_S	−	V-P	−	Rhamnose	+	Arginine	−
Cell shape	Short rods	Glucose	+	Citrate	−	Sucrose	+		
Arrangement	Mono and few Streptobacilli	Mannitol	+	TDA	+	Lactose	+		

R, Result; ONPG, Hydrolysis of o-nitrophenyl-ß-d-galactopyranoside; (ONPG) by action of ß-galactosidase and VP test, Voges-Proskauer test.

### Effect of different optimization strategies on BC production

#### Preoptimization of BC production by OVAT

**Figure 3:**
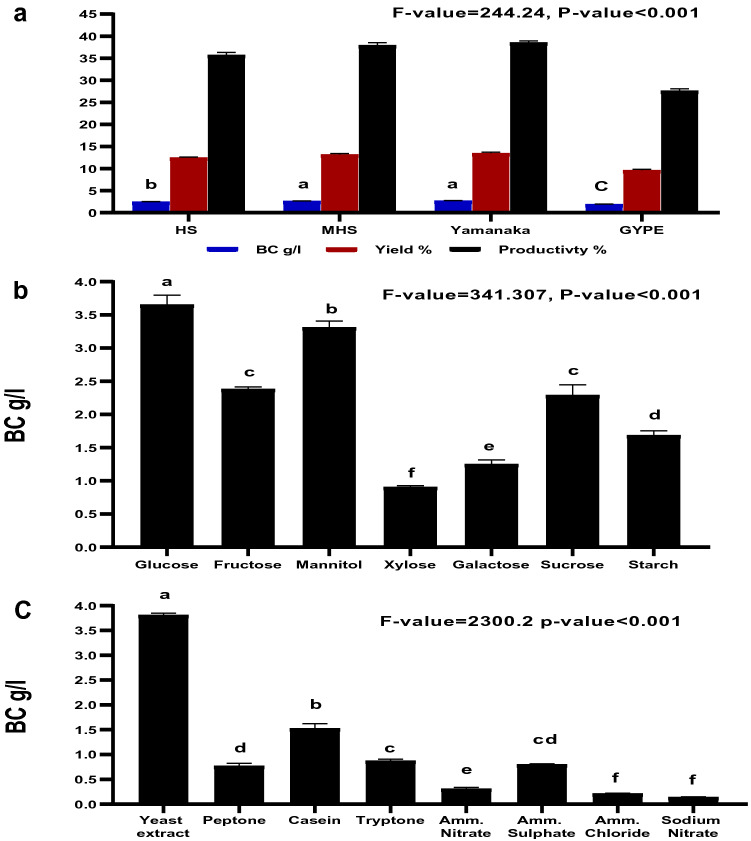
Effect of different media (**a**), carbon sources (**b**) and nitrogen sources (**c**) on the BC production by *L. plantarum* AS.6. Different letters above histogram are significantly different according to least significant difference test at p < 0.05.

Many factors influence BC production including the strain, medium composition, and other cultivation conditions^47^. Medium composition accounts for > 30% of BC production cost^48^. Accordingly, different standard BC production media were screened to identify the medium supporting the highest BC production for use as a basal medium for subsequent optimization processes. The results (Fig. 3a) revealed that three media supported similar BC production levels with a slight advantage in yield of BC using modified Yamanaka medium (2.7 g/l) over GYPE medium (1.93 g/l) (Fig. 3a). Complete consumption of media-glucose content was observed in all applied media with the highest BC yield and productivity of 13.5% and 38.6%, respectively, using modified Yamanaka media. In the previous work, the same medium supported the highest BC production using *Komagataeibacter hansenii* AS.5^33^. The effect of different carbon sources upon BC production was evaluated by the OVAT approach through substituting the original carbon (glucose) in the modified Yamanaka medium with other alternatives at 2% concentration. Results revealed that all of the carbon sources applied positively enhanced different ratios of BC production, whereas maximum BC production was attained through glucose 3.61 g/l (yield 18% and productivity 40.1%) as showed in Fig. 3b. It was reported that the carbon source is the main precursor for BC biosynthesis using different complex enzymes^49^. Different organic and inorganic nitrogen sources were also screened for BC production at 0.5% concentration. Complex-organic nitrogen sources were better than inorganic ones for supporting BC production, which is in agreement with other studies^49,50^. Yeast extract supported the highest BC production of 3.84 g/l (yield 19.2% and productivity 42.6%), followed by casein 1.53 g/l, whereas other nitrogen sources supported low to very low BC production as showed in Fig. 3c. The low capacity for most inorganic nitrogen sources in supporting high BC production is consistent with previous studies^50,51^. This could be attributed to the toxic effect and high ionic strength of inorganic nutrients^52^. Based upon the OVAT results, a modified Yamanaka medium, with glucose (carbon source) and yeast extract (nitrogen source), was used as the starting point for the next optimization process.

#### Screening of the independent variables using PBD

**Figure 4:**
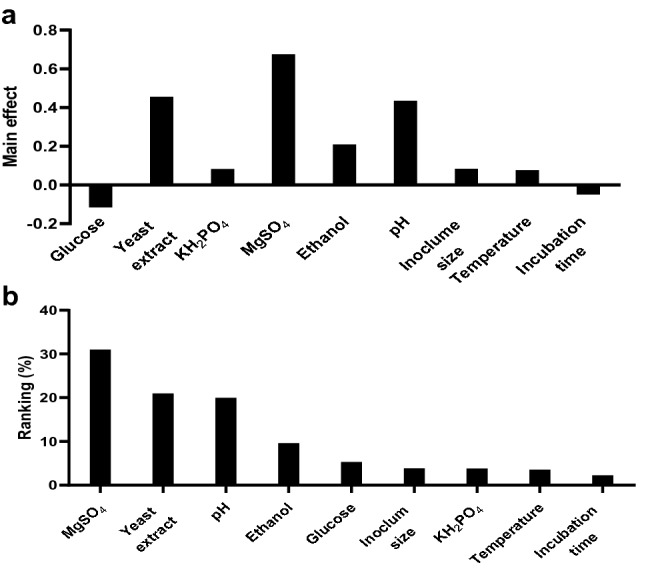
PBD results representing (**a**) The direct impact of the studied variables (in range of -1 to 1 for each variable as defined in Table 2), upon BC production, where (**b**) Pareto chart representing the relative ranking (%) of the magnitude of the culture conditions upon total BC production (100%).

According to the OVAT results, nine independent nutritional and physical variables were screened in 14 trials using PBD to evaluate effects on BC production. Each variable was tested in two levels coded (+ 1) and (− 1) for maximum and minimum values, respectively, where the produced BC (g/l) was measured as the design response (dependent variable). The results presented in Table 2 revealed a broad variation in the production of BC ranging from 1.27 g/l (Trial 3) to 4.07 g/l (Trial 11), signifying the impact of the optimization process upon BC production. The main effect results illustrated that among applied variables the yeast extract, KH_2_PO_4_, MgSO_4_, ethanol, pH, inoculum size, and temperature all contributed positively, where glucose and incubation time contributed negatively to BC production as shown in Fig. 4a. In the scope of this experiment, yeast extract, MgSO_4_, and pH were the most important variables affecting BC production as determined in the Pareto chart (Fig. 4b). The positive effect of yeast extract in BC production is likely to be due to the high nitrogen content and abundant growth factors in the yeast extract formula^53^. Several studies reported that pH values variation (between 4 and 9), affect BC production. This is related primarily to the producing strain used^54,55^, wherein the most commonly reported optimal pH was between a neutral and slightly-acidic pH range^28,56^. PBD rely on a first-order structural model, which according to regression analysis could be represented as following Eq. 1:

1 $$\begin{aligned} {\text{Y }}\left( {{\text{BC g}}/{\text{l}}} \right) & = { 2}.{23 } - \, 0.{\text{114X}}_{{1}} + \, 0.{\text{46X}}_{{2}} + \, 0.0{\text{82X}}_{{3}} \hfill \\ & \quad + \, 0.{\text{68X}}_{{4}} + \, 0.{2}0{\text{9X}}_{{5}} + \, 0.{\text{435X}}_{{6}} + \, 0.0{\text{83X}}_{{7}} \hfill \\ & \quad + \, 0.0{\text{ 63X}}_{{8}} - \, 0.0{\text{49X}}_{{9}} \hfill \\ \end{aligned}$$

**Table 2:** PBD for studied nine variables with coded, real values and response (estimated and predicted) for their contribution to enhanced BC production.

Trials	Variables*									Response	
	Glucose (X_1_)	Yeast extract (X_2_)	KH_2_PO_4_ (X_3_)	MgSO_4_ (X_4_)	Ethanol (X_5_)	pH (X_6_)	Inoculum Size (X_7_)	Temperature (X_8_)	Incubation Time (X_9_)	Estimated BC g/l	Predicted BC g/l
1	− 1 (15)	1 (7)	− 1 (2)	1 (0.05)	1 (7)	1 (7)	− 1 (9)	− 1 (20)	− 1 (8)	3.80	3.92
2	− 1 (15)	− 1 (3)	− 1 (2)	1 (0.05)	− 1 (3)	− 1 (3)	1 (11)	− 1 (20)	1 (10)	1.78	1.79
3	1 (25)	1 (7)	1 (4)	− 1 (0)	− 1 (3)	− 1 (3)	1 (11)	− 1 (20)	− 1 (8)	1.27	1.35
4	1 (25)	− 1 (3)	− 1 (2)	1 (0.05)	− 1 (3)	1 (7)	1 (11)	1 (30)	− 1 (8)	2.68	2.68
5	− 1 (15)	1 (7)	1 (4)	1 (0.05)	− 1 (3)	− 1 (3)	− 1 (9)	1 (30)	− 1 (8)	3.06	2.95
6	− 1 (15)	− 1 (3)	1 (4)	− 1 (0)	− 1 (3)	1 (7)	− 1 (9)	1 (30)	1 (10)	1.34	1.46
7	− 1 (15)	1 (7)	− 1 (2)	− 1 (0)	1 (7)	− 1 (3)	1 (11)	1 (30)	1 (10)	1.92	1.92
8	1 (25)	1 (7)	− 1 (2)	− 1 (0)	− 1 (3)	1 (7)	− 1 (9)	− 1 (20)	1 (10)	1.94	1.83
9	− 1 (15)	− 1 (3)	1 (4)	− 1 (0)	1 (7)	1 (7)	1 (11)	− 1 (20)	− 1 (8)	2.10	1.99
10	1 (25)	− 1 (3)	− 1 (2)	− 1 (0)	1 (7)	− 1 (3)	− 1 (9)	1 (30)	− 1 (8)	0.71	0.71
11	1 (25)	1 (7)	1 (4)	1 (0.05)	1 (7)	1 (7)	1 (11)	1 (30)	1 (10)	4.07	4.076
12	1 (25)	− 1 (3)	1 (4)	1 (0.05)	1 (7)	− 1 (3)	− 1 (9)	− 1 (20)	1 (10)	1.97	1.98
13	1 (25)	− 1 (3)	1 (4)	1 (0.05)	1 (7)	− 1 (3)	− 1 (9)	− 1 (20)	1 (10)	2.02	1.98
14	1 (25)	− 1 (3)	1 (4)	1 (0.05)	1 (7)	− 1 (3)	− 1 (9)	− 1 (20)	1 (10)	1.94	1.98

Variables*: X_1_–X_9_ (variable); the coded low and high level are presented as − 1 and 1, respectively. Variable levels are presented between brackets expressed as g/l for X_1_–X_4_; ml for X_5_- X_7_-; °C for X_8,_ and days for X_9_.

For this model, the values of *R*^2^ and adj-*R*^2^ for regression analysis express the accuracy of the applied model statistically such that values close to 1 correspond to high accuracy of the model^53^. In this experiment, the *R*^2^ and adj-*R*^2^ values were 0.993 and 0.976, indicating a high accuracy of the applied model. In the same regard, the significance *F* function indicates the significance of a given variable on the design results. The significance *F* value was 0.000651, indicating a high significance response. The Pareto chart is an absolute ranking for PBD results and revealed that yeast extract (*P-value* < 0.001), MgSO_4_ (*P-value* < 0.001), and pH (*P-value* < 0.001) represented the most significant variables affecting the BC production. These were studied further using BBD.

#### Optimization of the independent variables using BBD

**Figure 5:**
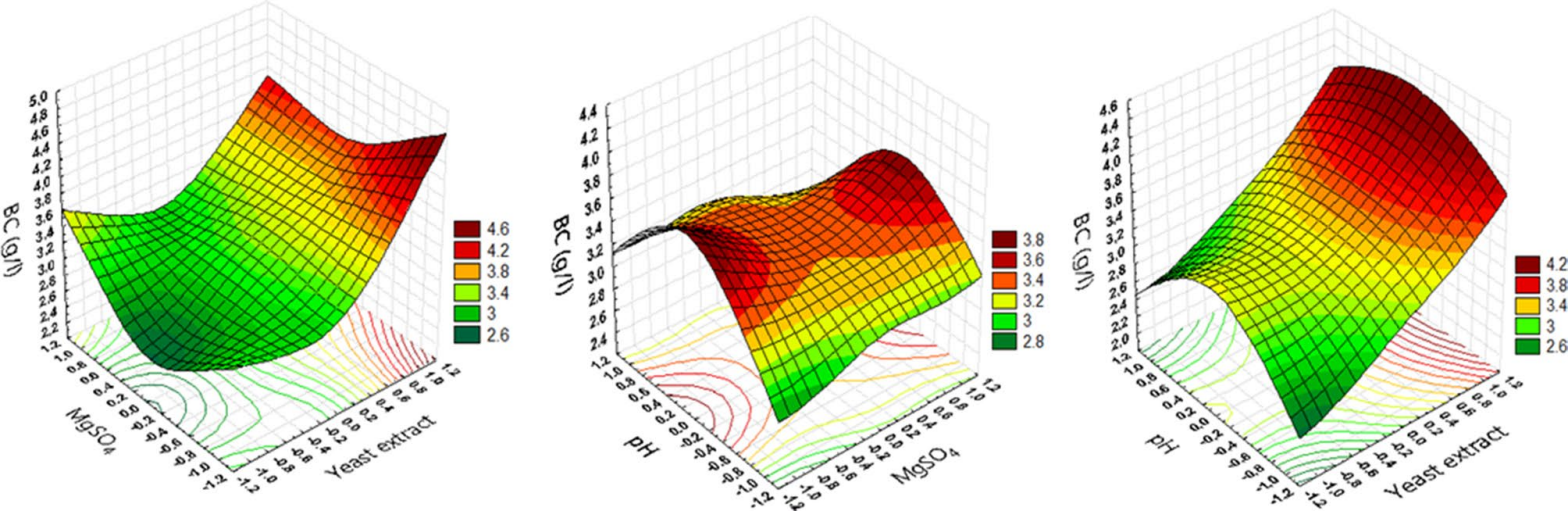
Three-dimensional surface plots representing the effect of yeast extract, MgSO_4_, and pH on the BC production by *L. plantarum* AS.6.

The most significant variables among PBD, yeast extract (X_1_), MgSO_4_ (X_2_), and pH (X_3_) were further studied using a three-level optimization design of BBD to identify the optimum levels for supporting maximal BC production. Currently, numerical designs, especially that of PBD, are widely accepted tools for the optimization of many biological processes^39,57,58^. The results presented in Table 3 revealed variations in BC production levels ranged from 2.6 to 4.3 g/l. The regression analysis results indicated that moving from a lower to a higher concentration of yeast extract and pH positively enhanced the BC production, contrary to MgSO_4_ wherein increasing the concentration adversely affected BC production. The effects of variable interactions on BC yield were represented using three-dimensional surface plots where every two variables were represented in the X-axes and BC production on the Y-axes, whereas the third variable remains constant at its middle value as shown in Fig. 5. The surface plots show that lower MgSO_4_ concentrations supported high BC production but that raising the yeast extract concentration resulted in higher BC production levels, particularly when the pH value was high and MgSO_4_ was low.

**Table 3:** BBD for selected four variables with coded and real values, observed and predicted results for BC production.

Trial	Variables*			Response	
	Yeast extract (X_1_)	MgSO_4_ (X_2_)	pH (X_3_)	Estimated BC g/l	Predicted BC g/l
1	− 1 (7)	− 1 (0.1)	0 (7)	3.09	3.01
2	1 (13)	− 1 (0.1)	0 (7)	4.32	4.38
3	− 1 (7)	1 (0.2)	0 (7)	3.32	3.27
4	1 (13)	1 (0.2)	0 (7)	3.87	3.95
5	− 1 (7)	0 (0.15)	− 1 (6)	2.59	2.69
6	1 (13)	0 (0.15)	− 1 (6)	3.76	3.71
7	− 1 (7)	0 (0.15)	1 (8)	2.67	2.71
8	1 (13)	0 (0.15)	1 (8)	3.85	3.76
9	0 (10)	− 1 (0.1)	− 1 (6)	3.01	3.01
10	0 (10)	1 (0.2)	− 1 (6)	3.21	3.17
11	0 (10)	− 1 (0.1)	1 (8)	3.25	3.29
12	0 (10)	1 (0.2)	1 (8)	2.95	2.96
13	0 (10)	0 (0.15)	0 (7)	3.38	3.31
14	0 (10)	0 (0.15)	0 (7)	3.33	3.31
15	0 (10)	0 (0.15)	0 (7)	3.202	3.31

Variables*: X_1_–X_3_ (selected variable to optimize their levels); the coded low, middle, and high levels are presented as − 1, 0, and 1, respectively. Variable levels are presented between brackets expressed as g/l for X_1_ and X_2_ and specific point for X_3_.

PBD regression results were fitted to a second-order polynomial structured model that relating the experiment response (BC production) to design variables as following Eq. 2:

2 $$\begin{aligned} {\text{Y}} & = {3}.{3}0{6}\, + \,0.{515625}\left( {{\text{X}}_{{1}} } \right)\, - \,0.0{4}0{62}\left( {{\text{X}}_{{2}} } \right)\, + \,0.0{1775}\left( {{\text{X}}_{{3}} } \right)\, \hfill \\ & \quad - 0.{17225}\left( {{\text{X}}_{{1}} {\text{X}}_{{2}} } \right)\, + \,0.00{65}\left( {{\text{X}}_{{1}} {\text{X}}_{{3}} } \right)\, - \,0.{127}\left( {{\text{X}}_{{2}} {\text{X}}_{{3}} } \right)\, \hfill \\ & \quad + 0.{229125}\left( {{\text{X}}_{{1}} } \right)^{{2}} \, + \,0.{116625}\left( {{\text{X}}_{{2}} } \right)^{{2}} \, - \,0.{31763}\left( {{\text{X}}_{{3}} } \right)^{{2}} , \hfill \\ \end{aligned}$$

where X_1_, X_2_, and X_3_ represent the three independent variables: yeast extract, MgSO_4_, and pH, respectively. At the model level, the very low significance *F* value in this experiment (0.001048) indicates the direct effect of the applied variables upon the measured response. The coefficient of determination (*R*^2^) was 0.979, indicating that 97.9% of the variation in BC yield was elucidated by the applied variables. An additional guide for the design accuracy is the adjusted coefficient of determination (Adj-*R*^2^) value, which was 0.943, indicating that the design predicted response fits well to the real measured response^58^. Additionally, the difference between *R*^2^ and Adj-*R*^2^ values was not significant, which demonstrates the fitness of the model for applied variables that guaranteed a maximum BC production. The MS *Solver* function was applied to the previous model to predict the exact levels of the studied variables supporting the maximum BC production and revealed that by using yeast extract of 13 g/l, MgSO_4_ of 1 g/l, and pH 7.2, the expected BC yield would be 4.4 g/l. This BC production level (4.4 g/l) in the optimized medium is 1.63 fold greater than that attained in the basal modified Yamanaka conditions. Similarly, BBD has also been applied to enhance BC production by *Komagetobacter intermedius*^50^, wherein the maximum BC yields attained was 3.906 g/l under optimized conditions. The applicability and accuracy of the applied model were verified in the laboratory under optimized conditions. The measured BC yield was 4.51 g/l, wherein the model predicted one was 4.4 g/l, indicating 98% accuracy of the applied model under the resulting optimal conditions: 15 (g/l) glucose, 13 (g/l) yeast extract, 1 (g/l) MgSO_4_, 4 (g/l) KH_2_PO_4_, 7 (ml/l) ethanol, at pH 7.2, with a cultivation temperature of 30 °C, incubation time of 8 days, and inoculum size of 11%. Under the final optimized media, the BC production by *L. plantarum* AS.6 was found to be 4.51 g/l, whereas reference strain *Gluconacetobacter hansenii* ATCC 23769 produced 2.85 g/l under the same optimization strategies^42^. Gram- positive and Gram-negative bacteria as BC producers were compared with *L. plantarum* AS.6 to explain their effectiveness on BC production. For Gram- positive bacteria, the productivity by *L. plantarum* AS.6 was 56% compared to the productivity by *Lactobacillus hilgardii* IITRKH159 (45%)^26^, *Lactobacillus acidophilus* (13%)^59^, *Leifsonia* sp (26%)^31^ and *Bacillus velezensis* SMR (52%)^60^. For Gram- negative bacteria, the productivity of *L. plantarum* AS.6 was compared to the productivity by *Komagataeibacter rhaeticus* K3 (36%)^61^, *Gluconacetobacter xylinus* FC01(28%)^43^, *Gluconacetobacter xylinus* ATCC 700178 (20%)^62^ and *Acetobacter xylinum* BPR 2001 (15%)^63^. The difference in the BC production of listed microbes might be due to several factors like carbon source, nitrogen source, strain type, cultivation time and optimization strategies, etc.

### Characterization of BC and BC/SA fabricated membranes

#### SEM analysis

**Figure 6:**
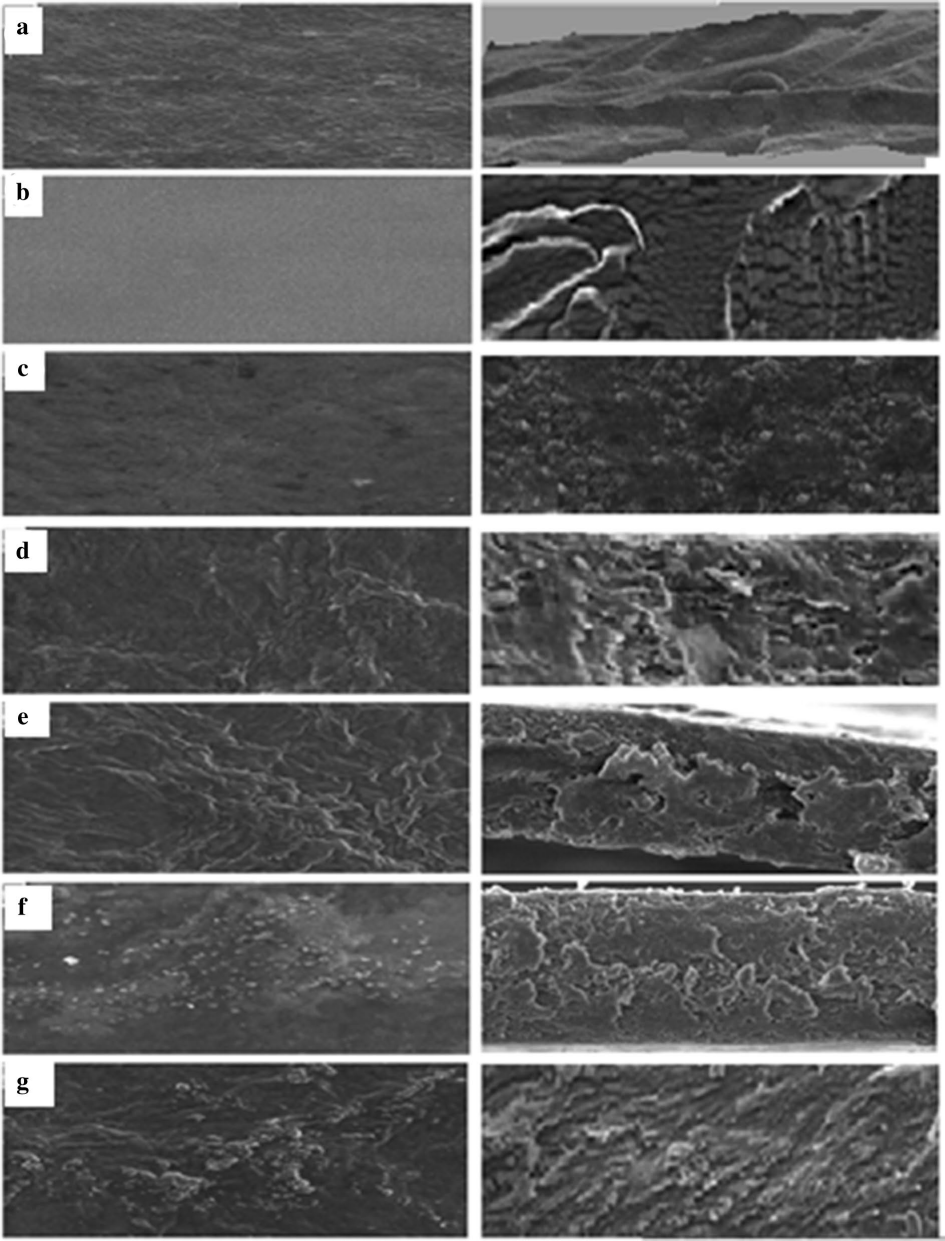
SEM images of surface morphology (right) and their corresponding cross-sectional (left) of (**a**) BC, (**b**) SA, (**c**) BC/SA, (**d**) BC/SA/GM1, (**e**) BC/SA/GM2, (**f**) BC/SA/GM3, and (**g**) BC/SA/GM4.

Firstly, the morphological structures of the dried BC, SA, and BC/SA composites were investigated using SEM and presented as surface and cross-section images. The BC membrane obtained from L. plantarum AS.6 is a purified, porous, and heterogeneous network structure (Fig. 6a). This densely packed, three-dimensional, and interconnected network originates from randomly arranged nano-and microcellulosic fibers^22,28^. Contrarily, the surface structure of SA film (Fig. 6b) appeared as a very smooth, nonporous, and homogeneous mat^14^. In the fabricated BC/SA composites, the network structure of BC was clearly dispersed (Fig. 6c) due to the homogenization and ultrasonic effects on the BC fibers from the fabrication process^64^. On BC/SA/GM composites, the GM appeared as white dots randomly distributed on the surface of BC/SA composites Fig. 6d–g. These white dots increased by increasing the GM concentration from 0.025 to 0.100 mg, indicating the applicability of the prepared composites (BS/SA) as antibiotic carriers especially in the case of GM.

#### Fourier transform-infrared spectroscopy (FT-IR) analysis

**Figure 7:**
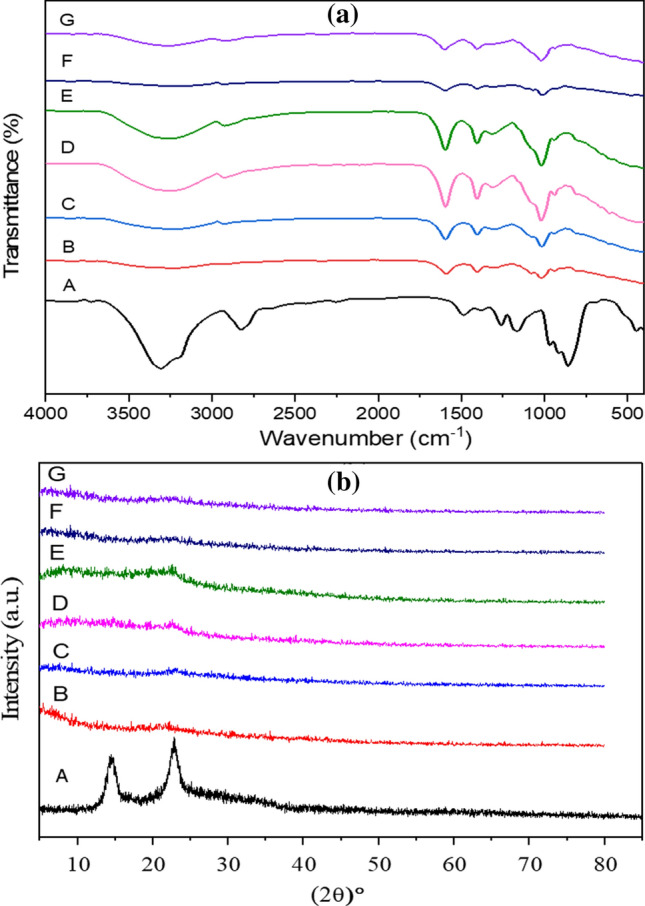
FT-IR (**a**) and XRD spectra (**b**) of [BC (A), SA (B), BC/SA (C), BC/SA/GM1 (D), BC/SA/GM2 (E), BC/SA/GM3 (F), and BC/SA/GM4 (G)].

Next, the chemical structure (functional groups and molecular bonding) of the BC, SA, and BC/SA as well the interaction between BC/SA and BC/SA/GM composites were evaluated by FT-IR spectroscopy (Fig. 7a). In the case of BC (Curve A), the band of intense absorption in the BC spectrum at 3354 and 2897 cm^−1^ were attributed to the presence of –OH group of cellulose type I and CH_2_ stretching vibrations, respectively, that are common for BC^22,65,66^. The cellulose absorption spectrum is the band at 1645 cm^−1^, which has been assigned to the –COOH group. In the case of SA (Curve B), the bands at 3212 cm^−1^ corresponding to –OH stretching, 1591 cm^−1^ to COO– asymmetric stretching, and 1404 cm^−1^ to COO– symmetric stretching^14^. In the case of BC/SA (Curve C), the interaction between BC and SA could be identified by the carbonyl and carboxyl group bands present in the range of 1900–1500 cm^−1^. The band of –OH was shifted to 3235 cm^−1^, with no new peaks detected^64^. In the case of BC/SA/GM (Curves D, E, F, and G), the bands of OH were shifted to high intensities compared with BC/SA. In addition, the peak intensities of OH increased with the increase of GM fabricated in the composites, whereas the other bands were approximately the same as in the BC/SA (Fig. 7a).

#### X-ray diffraction (XRD) patterns

The XRD patterns of BC membrane, BC/SA, and BC/SA/GM composites are presented in Fig. 7b, The characteristic pattern of XRD display typical diffraction peaks of the crystalline structure of type I crystalline cellulose with the most prominent peak located at 2Ѳ = 23.4° and two overlapping peaks at 2Ѳ = 15.2° and 17.5°, whereas the intensities of the characteristic crystal peaks were lower in BC fabricated composites. This is due to the long fibrils of BC, which blend and homogenize to form a slurry of shorter fibers of BC that partially destroy the beta-1,4 glycosidic bonds and hydrogen bonds polymer of D-glucose units, resulting in lower crystallinity of fabricated BC^14,67^. The amorphous structure of SA was observed from the XRD pattern. All fabricated BCs exhibit little changes for XRD pattern after being incorporated with different GM concentrations (Fig. 7b).

#### Mechanical properties

Different mechanical properties of the BC and fabricated BC/SA membranes were evaluated in terms of maximum load, tensile strength, elongation, Young’s modulus, and thickens of the dried samples (Table 4). The results indicated that the BC membrane revealed higher mechanical features compared with fabricated composites, which could be attributed to the homogenous and random BC fibers networking with strong hydrogen bonds^67,68^. Contrarily, the applied BC fabrication process involved BC fibers blending that negatively affects the mechanical properties of the resulting composites. The results revealed slight improvement in the mechanical properties of the BC/SA composites compared with that of the sole SA, which is consistent with a previous study^14^. Increasing the GM concentration in BC/SA composites, directly reflected on the mechanical properties improvement, this finding may be attributed to the penetration of GM particles into BC, giving rise to a dense network on the BC microfibrils (Table 4), likewise, GM (1–5% wt.) was also reported to enhance the mechanical properties of chitosan-alginate composites^69^. The significant Young’s modulus and tensile strength reported for *L. plantarum* AS.6 BC in the current study nominated it as scaffold for various applications: including food packaging, bone tissue engineering and artificial heart valve manufacturing. In addition the effect of the fabrication conditions with sodium alginate greatly enhanced the BC membrane in term of elasticity and stretching (reduction in the Young's modulus with higher elongation), a prerequisites for membrane compatibly for wound dressing applications^70,71^.

**Table 4:** Mechanical properties of BC membrane and BC fabricated composites.

Sample (%)	Maximum load (N)	Tensile strength (MPa)	Elongation (%)	Young’s modulus (MPa)	Thickens (mm)
BC	7.75	7335	1.66	7838	0.25
SA	4.04	2122	8.18	6095	0.14
BC/SA	4.82	6502	4.74	6366	0.15
BC/SA/GM1	2.59	3050	5.42	2576	0.15
BC/SA/GM2	4.09	3441	6.03	3141	0.15
BC/SA/GM3	5.08	4612	6.42	5484	0.16

#### Antimicrobial assay

**Figure 8:**
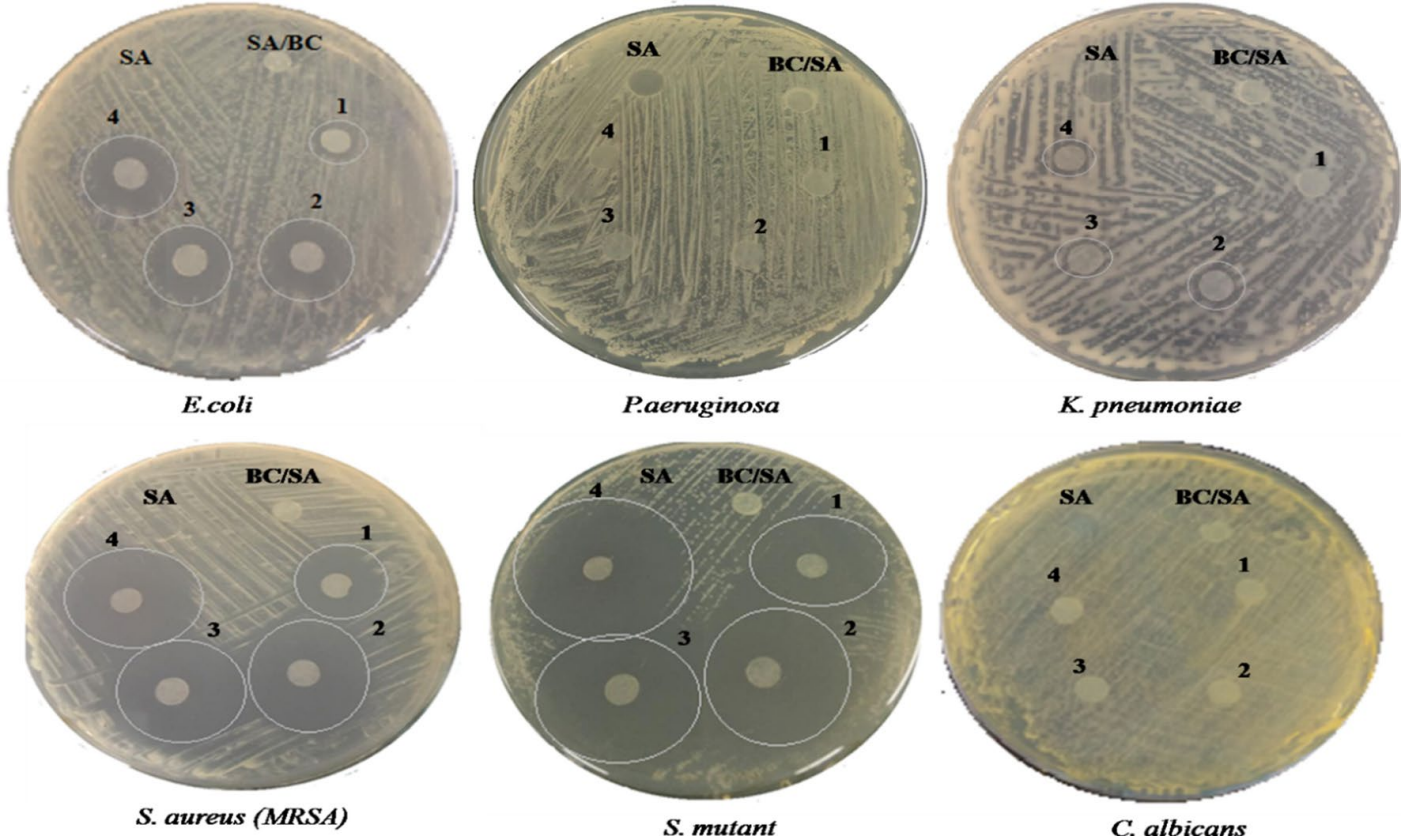
Disk diffusion method for antimicrobial activity expressed as halo zones of the prepared BC/SA/GM composite at four GM concentrations 1 (0.025), 2 (0.050), 3 (0.075), and 4 (0.100) against six pathogenic microorganisms.

Lastly, the antimicrobial activity of a prepared composite of BC/SA at different concentrations of GM was evaluated using the disk diffusion method. As shown in Table 5 and Fig. 8, the SA and BC/SA composites have activity on of the model pathogens applied. The addition of GM to BC/SA composites enhanced the antimicrobial activity against all of the model microbes except for *P. aeruginosa* and *C. albicans*. These results indicated increasing the GM concentration was correlated with increased antibacterial activity of BC/SA composites against *E. coli*, *S. mutans*, *S. aureus*, and *K. pneumoniae*. Based on the diameters of inhibition zones (Table 2), the highest antimicrobial activities were against *S. mutant*, *S. aureus*, *E. coli*, *and K. pneumoniae,* in order. The varied antimicrobial activity (Different halo zones) may be attributed to differences in the susceptibility of different microbes to prepared composites^72^. GM exerts its action through inhibiting the protein synthesis, nucleic acid reproduction, formation of vital metabolites, and DNA destruction of bacteria that lead to the cell membrane rupture and subsequent death of cells^69,73^. Hence, the antimicrobial activity of the BC membrane is neglected^74,75^; several studies reported its fabrication with other materials to enhance this activity including BC/SA/silver sulfadiazine^72^, BC/SA/PHMB^76^, BC/SA/silver nanoparticles^77^, and BC/SA/chitosan/copper sulfate^78^. Combining all beneficial qualities would make prepared BC/SA/GM composites good antibacterial wound dressing materials as well as for other biomedical applications.

**Table 5:** The antimicrobial activity of the prepared BC/SA/GM composite at four concentrations against six pathogenic microorganisms.

Organism	Diameter of halo-zone (mm)					
	Samples					
	SA	BC/SA	BC/SA/GM1	BC/SA/GM2	BC/SA/GM3	BC/SA/GM4
*E. coli*	0	0	9 ± 0.85	14	15 ± 0.23	16 ± 0.84
*P. aeruginosa*	0	0	0	0	0	0
*K. pneumoniae*	0	0	0	8	9 ± 0.46	10 ± 0.78
*S. aureus*	0	0	20 ± 0.51	28 ± 0.47	29 ± 0.58	31 ± 0.12
*S. mutant*	0	0	29 ± 0.12	35 ± 0.76	38	40 ± 1.49
*C. albicans*	0	0	0	0	0	0

## Conclusion

BC is an extra-polysaccharide with several promising applications, commonly distributed among Gram-negative bacteria. In the scope of this study, a locally isolated Gram-positive *Lactiplantibacillus plantarum* strain AS.6 showed good potential for BC production*.* Use of different optimization approaches (conventional and statistical) resulted in a yield of 4.51 g/l BC under the following optimal conditions: 15 g/l glucose, 13 g/l yeast extract, 1 g/l MgSO_4_, 4 g/l KH_2_PO_4_, 7 ml/l ethanol, at pH 7.2, cultivated at 30 °C, for 8 days, with an inoculum size of 11%. The produced BC under optimal conditions (4.51 g/l) represent a two-fold increase in yield over the basal medium. Fabrication of produced BC with SA and GM revealed strong antibacterial activity against *Escherichia coli*, *Klebsiella pneumoniae*, *Staphylococcus aureus*, and *Streptococcus mutans*, indicating its good potential applicability for drug delivery and wound dressing applications. Overall, the newly fabricated BC/SA/GM composites could be applied as promising antibacterial wound dressing materials as well as in other biomedical applications. Future research should be directed to explore novel BC producers with the goal of reducing production cost.

## Materials and methods

### Isolation and screening of BC-producing bacteria

Rotten samples taken from bananas, apples, palms, guavas, oranges, tangerines, peaches, strawberries, dates, pineapples, lemons, tomatoes, and vinegar were used to isolate potential BC producers. Approximately 5 g or ml from each sample was mixed in 45 ml of Hestrin and Schramm medium with slight modification (MHS) in a 250 ml Erlenmeyer flask containing: 20 g/l d-glucose, 5 g/l peptone, 5 g/l yeast extract, 2.7 g/l Na_2_HPO_4_, 1.15 g/l citric acid, 2 ml acetic acid, 5 ml ethanol, and 2 ml Nystatin (100,000 I.U./ml). Then the flasks were incubated statically at 30 °C for 7 days. Flasks with a white pellicle covering the surface of the liquid medium at the air–liquid interface were selected as being positive for BC production. For enrichment, the white pellicle was transferred to 50 ml of HS medium under static cultivation at 30 °C for 7 days. The culture broth of the selected flask was serially diluted with 0.9% NaCl (w/v) (from 10^−1^ to 10^−10^ dilutions) and 100 µl of the diluted samples (10^−6^ and 10^−8^) were spread on Glucose Ethanol Yeast extract media (GEY) agar, which is composed of (g/l): 20 d-glucose, 10 yeast extract, 5 ml ethanol, 3 CaCO_3_, and 20 agar. The agar plates were incubated at 30 °C for 5 days. After incubation, colonies with a clear zone surrounding them were selected and inoculated in a test tube containing 5 ml of HS medium composed of (g/l):20 d-glucose, 5 peptone, 5 yeast extract, 2.7 Na_2_HPO_4_, 1.15 citric acid, 5 ml ethanol, and incubated at 30 °C for 48 h with shaking at 200 rpm as a preinoculum^79^. This was inoculated in 45 ml of HS liquid media and incubated statically at 30 °C for 7 days. Subsequently, the purified isolated colonies with a white pellicle on the surface of flasks were stored at 4 °C as BC-producing isolates for further study.

### Identification of the BC-producing isolate

#### Molecular identification

Molecular identification of the BC-producing isolate was performed by using 16S ribosomal RNA (16S rRNA) sequence analysis. Firstly, genomic DNA was extracted from pure culture by the salting-out method^80^. Secondly, the *16S rRNA* gene from the genomic DNA of the selected isolate was amplified by polymerase chain reaction (PCR), using universal degenerate primers designed to amplify the full length (1500 bp) of the *16S rRNA* gene according to the *E. coli* genomic DNA sequence. The forward primer was 5′ AGAGTTTGATCMTGGCTCAG-3′ and the reverse primer was 5′ TACGGYTACCTTGTTACGACTT-3′^81^. Thirdly, the amplified PCR product was purified to remove unincorporated nucleotides and excess primers using a PCR purification kit (QIAgen PCR Purification Kit). Fourthly, the purified PCR product was sequenced using the dideoxy chain termination method^82^. This was done using an ABI PRISM model 3730 automated DNA sequencer at Sigma for Scientific Research. Finally, the sequences were assembled using BioEdit Sequence Alignment Editor Program^83^, and comparative sequence analyses were performed using Clustal W. The BLAST program (http://blast.ncbi.nlm.nih.gov/Blast.cgi) was used to assess the similarity, and a neighbor-joining method was used for the construction of the phylogenetic tree by MEGA software version 4.0^84^.

#### Morphological and biochemical characterizations

The BC-producing isolate was subjected to further characterization to analyze morphological characteristics by SEM, Gram reactivity, and observations of the colony surface, configuration, margin, cell shape, and pigmentation. Biochemical characterization was carried out using an Oxoid commercial Kit (Microbact GNB 24E Kit) according to the manufacturer’s instructions with reference to Bergey’s Manual of Systematic Bacteriology.

#### Stander preparation of preinoculum

From BC-producing isolates, a freshly and pure single colony was cultured in HS medium and then incubated at 30 °C for 2 days under agitated condition (200 rpm) as the standard preinoculum preparation for further use.

#### Production, harvesting, purification, and parameters of BC

The production of BC was performed using the initial production HS as the standard media, at pH 5.5 with 10% inoculation from the preinoculum of the selected isolate, with static incubation for 7 days at 30 °C. The BC pellicle formed at the air–liquid interface was harvested from the culture medium and washed various times with distilled water (dH_2_O) to get rid of excess medium components. Afterward, the pellicle was then treated with 0.5% NaOH at 90 °C for 30 min, to remove bacterial contaminants and other impurities immobilized on the BC membrane, and then washed by dH_2_O until neutralization. Finally, the purified BC pellicle was dried at 70 °C overnight until reaching constant weight^85^. The dry weight (dry wt.), yield, and productivity of BC were recorded as parameters of BC production^26,43^. The BC was estimated as the dry wt. of BC and expressed as g/l, yield of BC % = (BC dry wt. g/l)/(Original sugar g/l) × 100 and productivity of BC % = (BC dry wt. g/l)/(Production time d) × 100.

#### Preoptimization experiments for BC production (OVAT)

To determine the best basal medium for BC production by the locally isolated *L. plantarum* AS.6, different standard BC production media were screened including HS media^79^, modified HS^86^, modified Yamanaka media^87^, and modified GEM^88^. The medium supporting the highest production was used as a basal medium for screening the effects of different carbon and nitrogen sources upon BC production by the OVAT method. Different carbon sources (2%), including glucose, fructose, mannitol, xylose, galactose, sucrose, and starch, in the presence of different organic/inorganic nitrogen sources (0.5%), including, yeast extract, peptone, casein, tryptone, ammonium nitrate, ammonium sulfate, ammonium chloride, and sodium nitrate, were surveyed to determine the best carbon and nitrogen source supporting the highest BC production. The production process was performed in 250 ml Erlenmeyer flasks containing 50 ml sterile production basal medium. The optimization experiments were conducted in triplicate at static conditions.

#### Screening of independent variables using PBD

PBD was applied to screen the influence of different nutritional and physical parameters upon BC production^40^. In the current study, nine independent variables were explored: glucose (X_1_), yeast extract (X_2_), KH_2_PO_4_ (X_3_), MgSO_4_ (X_4_), ethanol (X_5_), pH (X_6_), inoculum size (X_7_), cultivation temperature (X_8_), and incubation time (X_9_). These were screened in 12 different trials in addition to two central trials to evaluate the accuracy of PBD model. Each variable was tested in two levels coded (+ 1) and (− 1) for maximum and minimum values, respectively, where produced BC (g/l) was measured as the design response (dependent variable) (Table 3). Within the PBD matrix, each row represents an experiment where each column represents an independent variable (Table 3). The PBD results were fitted in the first-order polynomial equation (Eq. 1).

$${\text{Y}} = \upbeta_{0} + \sum \upbeta_{{\text{i}}} {\text{X}}_{{\text{i}}} .$$

In this model, Y represents the response (BC g/l), β_0_ is the model intercept, β_i_ is the variable estimate, and X_i_ represents the levels of the independent variables. The significance of variables was determined by calculating the *p*-value through standard regression analysis. All trials were prepared in triple flasks containing 50 ml of the corresponding medium according to the design matrix.

#### Optimization of the independent variables using BBD

The most significant variables identified from PBD were further studied using the three-level optimization design of BBD^89^ to identify the optimum levels for supporting maximum BC production. Application of BBD consisted of three main steps: performing the statistically designed experiments, analyzing the results to predict the targeted response, and finally checking the adequacy of the applied model. A total of 13 full factorial BBD trials plus two central extra trials (sum 15 trials) were used to investigate the individual and synergetic effects of the three selected variables, identified through PBD, upon BC production. The significant variables according to PBD results were yeast extract (X_1_), MgSO_4_ (X_2_), and pH (X_3_). Each variable was studied at three different levels (− 1, 0, + 1), where 0 represents the central value of each variable and + 1 and − 1 represent the high and low value of each variable, respectively (Table 3). The other medium components and cultivation parameters were kept at their effective levels (positive or negative) according to the regression analysis of the PBD experiment. The experimental results were fitted by regression to a predictive quadratic polynomial that anticipates the BC production (Y) as a function of culture conditions (X). The results were fitted to the following second-order polynomial structured model for three variables Eq. 2:

$$\begin{aligned} {\text{Y }} & = {\text{b}}_{0} + {\text{b}}_{{1}} \left( {{\text{X}}_{{1}} } \right) \, + {\text{b}}_{{2}} ({\text{X}}_{{{2})}} + {\text{b}}_{{3}} \left( {{\text{X}}_{{3}} } \right) + {\text{b}}_{{{12}}} ({\text{X}}_{{1}} {\text{X}}_{{{2})}} + {\text{b}}_{{{13}}} \left( {{\text{X}}_{{1}} {\text{X}}_{{3}} } \right) \, \hfill \\ & \quad + {\text{b}}_{{{23}}} \left( {{\text{X}}_{{2}} {\text{X}}_{{3}} } \right) \, + {\text{b}}_{{{11}}} \left( {{\text{X}}_{{1}} } \right)^{{2}} + {\text{b}}_{{{22}}} \left( {{\text{X}}_{{2}} } \right)^{{2}} + {\text{b}}_{{{33}}} \left( {{\text{X}}_{{3}} } \right)^{{2}} \hfill \\ \end{aligned}$$

Y is the predicted response (BC production); β_0_ is constant; β_1_, β_2_, and β_3_ are linear coefficients; β_12_, β_13,_ and β_23_ are cross-product coefficients; and β_11_, β_22,_ and β_33_ are quadratic coefficients, Eq. (2) was used to predict the optimum levels of the selected independent variables by setting the partial derivative with respect to each independent variable to zero. All experiments were performed in triplicate.

#### Data analysis

The data of BC production (g/l) was subjected to multiple-linear regressions using MS Excel 2007 to estimate *t*-value, *p*-value, and confidence levels. The significance level (*p*-value) is determined using the Student’s *t*-test. The confidence level is an expression of the *p-*value in percent. Optimal production values for each independent variable were predicted using the “Solver” function of MS Excel 2007. The results of BBD were visualized in form of three-dimensional surface plots using STATISTICA 7 software.

#### Fabrication of BC/SA/GM composites

**Figure 9:**
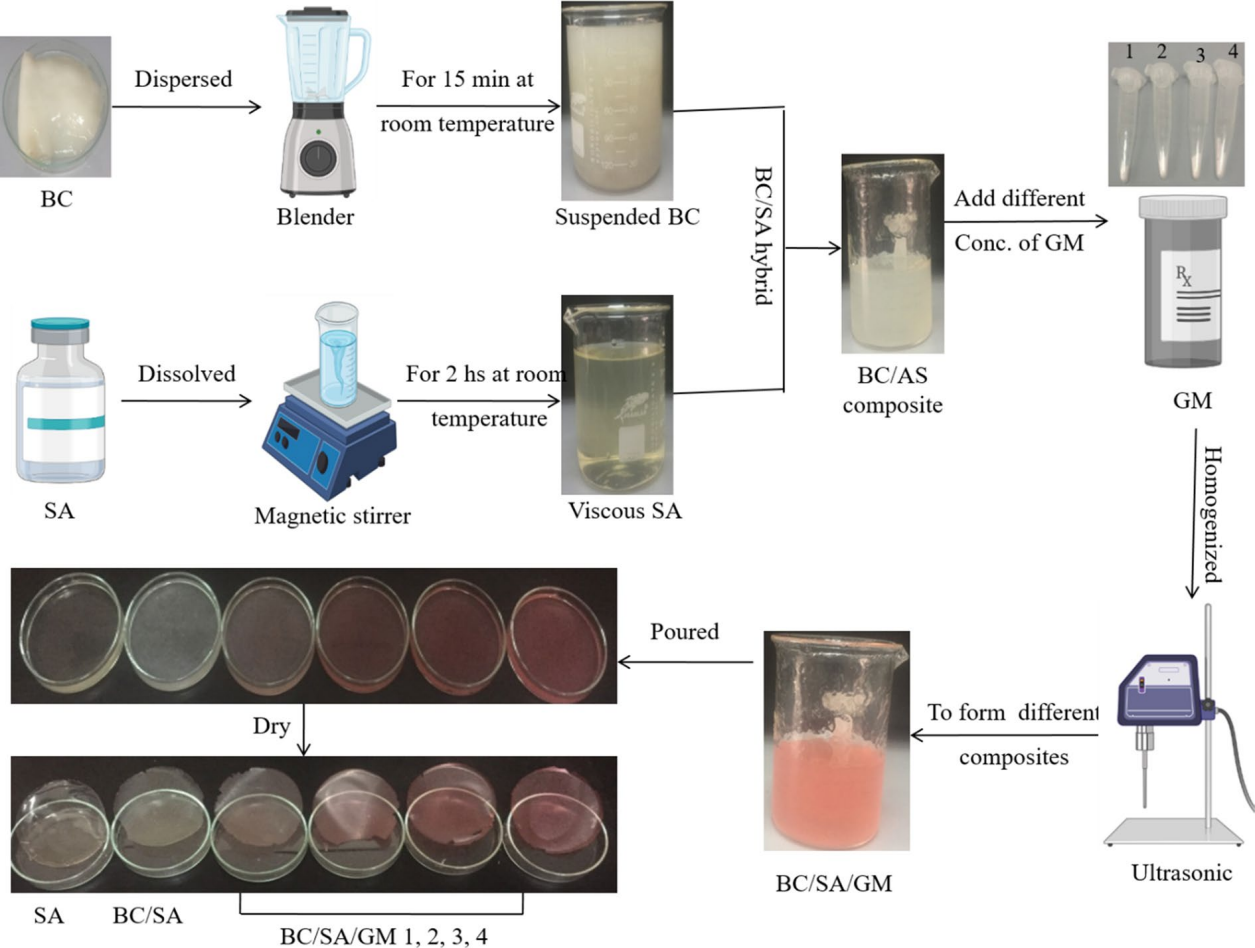
Schematic fabrication process of BC/SA composite membranes.

A simple solution mixing and casting approach was used to prepare the BC/SA/GM composites. The preparation procedure is divided into three steps. Firstly, 50 g of wet BC were cut into small pieces and dispersed in 100 ml dH_2_O by a high-speed blender (800ES blender, 230 V, 50 HZ, 330 W, model BB90E, USA) for 15 min at room temperature to form a BC suspended fiber. Secondly, SA at 2% (w/v) was dissolved in dH_2_O at room temperature to form a viscous solution. To prepare the BC/SA hybrid composite, both BC and SA solutions were mixed at a ratio of 2:3 to obtain homogenous BC/SA dispersions. Thirdly, GM at different concentrations (0.025, 0.050, 0.075, and 0.100 mg) was added to the 25 ml of BC/SA dispersions, homogenized by ultrasonication (750 wt, 20 KH, pulse 45, Amp 1) for 5 min under ice-water bath, and two drops of glycerol added as a stabilizing agent (labeled as BC/SA/GM1, BC/SA/GM2, BC/SA/GM3, and BC/SA/GM4). The mixture of each concentration was poured into a 6 cm Petri dish and left to dry in an oven at 40 °C for 48 h. The BC/SA and SA membranes free from GM were used as control. The membranes were carefully peeled off from the plates and were stored in a vacuum desiccator until further use. The procedure for the fabrication of composite membranes is shown in Fig. 9.

#### Characterization of BC and BC/SA fabricated membranes

The morphological structures of BC and BC fabricated composites were investigated by using SEM (JEOL JSM 6360 LA, Japan). The dried samples were firstly precoated with a thin layer of gold nanoparticles to reduce charge interruptions during SEM imaging. The chemical groups, stretching vibrations, and band intensities of the samples were examined using FT-IR spectra (Shimadzu FTIR-84 00 S, Japan). For each sample, the spectra were recorded at room temperature in a range of 4000–500 cm^−1^, at a resolution of 1 cm^−1^ with baseline corrections. The samples’ crystallinity was examined through an XRD (XRD, X PERT PRO-PAN Analytical- Netherlands) with a back monochromatic and a Cu anticathode. The samples were scanned with 2θ ranging from 5° to 80° at a scan rate of 5°/min. The mechanical properties of the dried samples were determined by using a universal tensile testing machine (Universal Testing Machine, model: AG-I/50 N-10 kn, Japan) operating at a crosshead speed of 2 mm/min at room temperature. The samples were cut into rectangular strips (5 by 1 cm) for measurement, with a gauge length of 20 mm. An electronic digital micrometer was used to determine the samples’ thickness before the examination. Three specimens were tested and average results were reported.

#### Antimicrobial activity of BC/SA/GM composite membranes

The antimicrobial activity of the composite BC/SA loaded with four different concentrations of GM (0.025, 0.050, 0.075, and 0.100 mg) were investigated qualitatively by the disk diffusion approach against six pathogenic reference strains: *Escherichia coli* (ATCC 25922), *Pseudomonas aeruginosa* (ATCC 27853), and *Klebsiella pneumoniae* (ATCC 13883) as Gram-negative bacteria; *Staphylococcus aureus* (ATCC 25923) and *Streptococcus mutans (*ATCC 25175) as Gram-positive bacteria; and *Candida albicans* (ATCC 10231) as a yeast model. The disk diffusion approach was performed in a Petri dish containing Luria–Bertani (LB) medium, with disks (5 mm) of each composite membrane. Cultures of tested pathogens were grown in LB broth at 37 °C and 200 rpm for 24 h. After incubation time, 100 μl of serially diluted pathogens (10^6^ CFU/ml) were inoculated separately on LB agar along with BC/SA disks loaded with different GM concentrations and incubated at 37 °C for 24 h^90^. The resulting inhibitory actions of tested membrane disks were assessed by measuring the inhibition diameter (Halo zone including composite disks). The GM-free membranes of BC/SA and SA were used as a control. The composite membranes were sterilized through UV light for 60 min^72,91^. The results represented are the average values of triplicate experiments.
